# Focal peliosis hepatis with undefined etiology:: Diagnostic elucidation via ultrasonography, CT, and MRI; case report with literature review

**DOI:** 10.1097/MD.0000000000044151

**Published:** 2025-12-19

**Authors:** Tingting Geng, Sumei Gao, Olena Kovalska, Zhijian Liu

**Affiliations:** a Department of Intensive Care Unit, Weifang People’s Hospital, Wei Fang, Shan Dong, China; b Department of Pathology, Weifang People’s Hospital, Wei Fang, Shan Dong, China; c Department of Radiology, Seychelles Hospital, Healthcare Agency, Victoria, Seychelles; d Department of Radiology, Weifang People’s Hospital, The First Affiliated Hospital of Shandong Second Medical University, Weifang, Shandong, China.

**Keywords:** computed tomography, magnetic resonance imaging, peliosis hepatis, tomography, ultrasonography, X-ray computed

## Abstract

**Rationale:**

Peliosis hepatis is an uncommon benign vascular disorder characterized by sinusoidal dilatation and multiple blood-filled cystic cavities within the hepatic parenchyma. Clinically, it is frequently asymptomatic and often detected incidentally. Its radiological manifestations are complex, highly variable, and lack specificity, leading to frequent misdiagnoses of primary hepatic tumors, metastatic lesions, or abscesses.

**Patient concerns:**

We present the case of a 23-year-old female who underwent ultrasound, computed tomography, and magnetic resonance imaging, revealing hepatic lesions. The patient had no history of liver disease or drug use.

**Diagnoses:**

The diagnosis of peliosis hepatis was confirmed via histopathology.

**Interventions:**

laparoscopic partial hepatectomy was performed.

**Outcomes:**

The patient recovered well during the outpatient follow-up.

**Lessons:**

Peliosis hepatis should always be recognized in the differentiation of liver lesions, especially in the presence of atypical imaging findings, without any history of liver-related diseases.

## 1. Introduction

Peliosis hepatis (PH) is a vascular disorder characterized by sinusoidal dilatation and multiple blood-filled cystic cavities. The exact etiology remains elusive, although reports in the literature suggest associations between pharmacological agents, infections, and malignancies. It is important to summarize the characteristic imaging features of focal PH for accurate diagnosis. This article reports a case of focal PH in a young woman without etiology combined with a systematic literature review to summarize the imaging features and provide clinicians with ideas for an accurate diagnosis.

## 2. Case presentation

A 23-year-old female presented with a hepatic hemangioma incidentally discovered during a routine physical examination 6 months prior to presentation. The patient reported no abdominal pain, distension, nausea, vomiting, chills, or fever and exhibited no jaundice in the skin or sclera. Her medical history was unremarkable, with no evidence of hypertension, diabetes mellitus, cardiovascular disease, hepatitis, tuberculosis, or other infectious diseases. She denied drug or food allergies, trauma, surgery, transfusion, occupational exposure to toxins, or familial genetic disorders.

### 2.1. Laboratory results

Routine blood work and liver and renal function tests were within normal limits: hemoglobin 120 g/L, white blood cell count 3.84 × 10⁹/L, platelets 174 × 10⁹/L, glucose 4.7 mmol/L, triglycerides 0.59 mmol/L, alanine aminotransferase 8U/L, aspartate aminotransferase 11U/L, alkaline phosphatase 51U/L, total bilirubin 12.6 µmol/L, total protein 65.9 g/L, albumin 40.9 g/L, creatinine 41µmol/L, uric acid 239 µmol/L. prothrombin time was normal. Serology for hepatitis B and C viruses, treponemal pallidum antibodies (TP-Ab), and human immunodeficiency virus antibodies (HIV-Ab) were negative. Tumor markers were within normal ranges: alpha-fetoprotein < 1.30ng/mL (0.00–7.00),carcinoembryonic antigen < 0.50 ng/mL(0–5), and carbohydrate antigen199 33.47 U/mL (0–34).

### 2.2. Imaging findings

**Figure 1.**
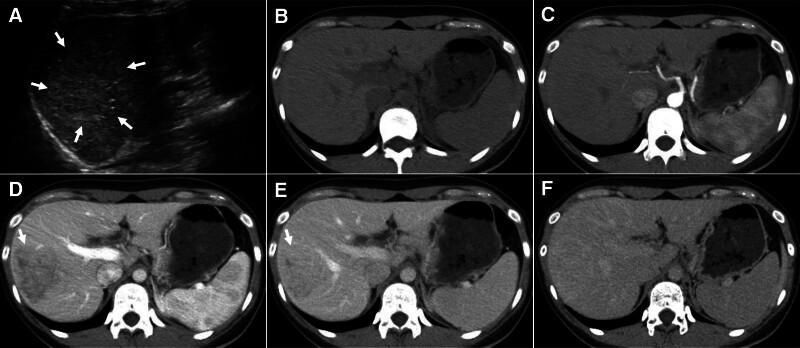
Ultrasonography and CT imaging of peliosis hepatis. (A) Ultrasonographic findings showing a slightly hyperechoic, ill-defined, round lesion in the right hepatic lobe (arrows). (B) Non-contrast CT showing no abnormal density within the liver. (C) Arterial phase demonstrating a patchy iso- to mildly hypodense lesion in segment VII of the right hepatic lobe. (D) Portal venous phase showing a heterogeneous hypodense lesion in segment VII (arrow). (E–F) Delayed phase with persistent centripetal enhancement, reduced lesion size, and ill-defined margins exhibiting isodensity (arrow).

**Figure 2.**
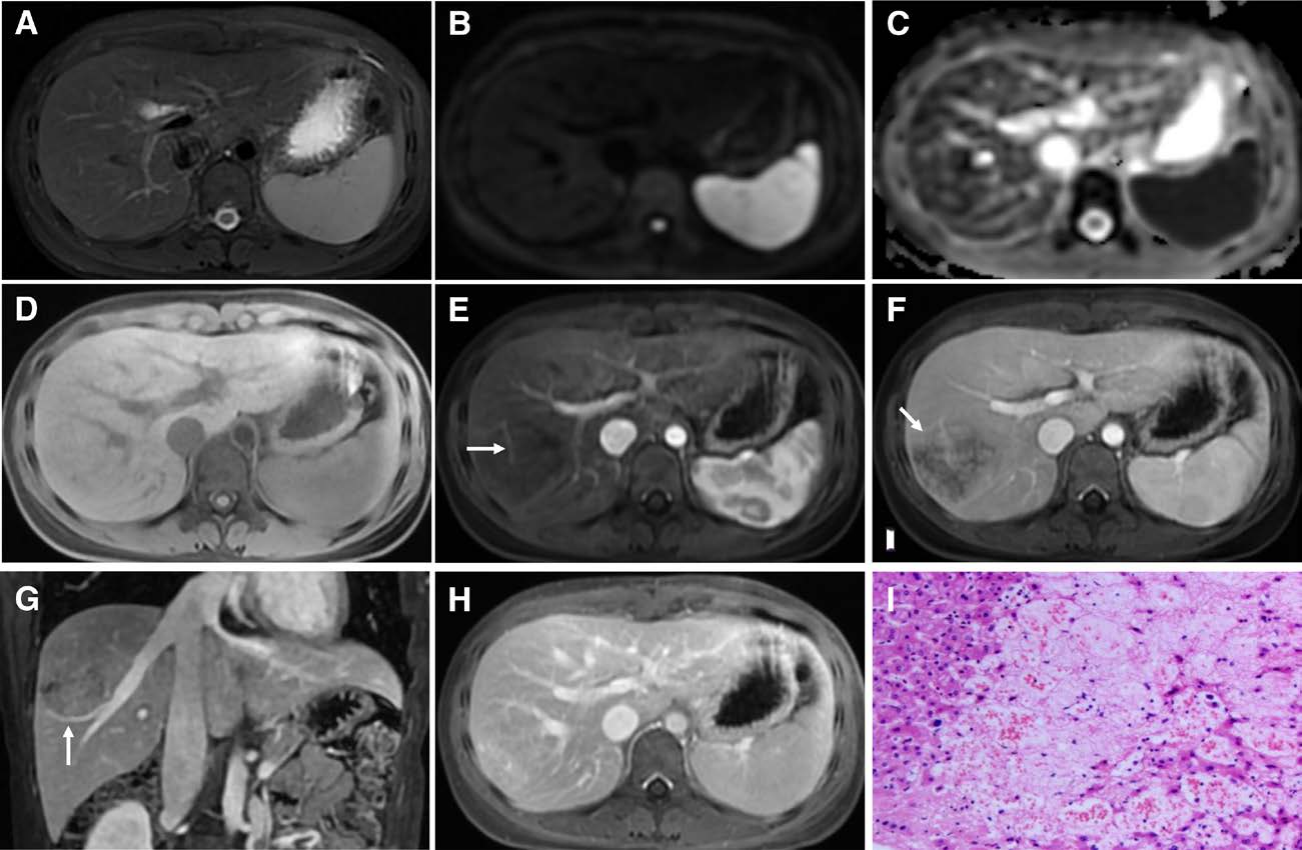
MRI imaging of peliosis hepatis and Pathological examination. (A-C) T2WI, DWI, and ADC maps showing isointense signals in the lesion. (D) T1WI revealing a heterogeneous slightly hypointense signal. (E–H) Post-contrast images, the lesion showing progressive centripetal enhancement from the arterial (E) to portal (F, arrow) and delayed (G;H) phases, with delayed-phase imaging displaying iso- to mildly hyperintense signals. Vascular structures traversing the lesion are visible (E, arrow), mild vascular displacement at lesion margins (G, arrow). (I) Multifocal sinusoidal dilation, widening, and hemorrhage accompanied by irregular capillary hemangioma-like proliferation (hematoxylin and eosin staining; magnification × 40). ADC = apparent diffusion coefficient, DWI = diffusion-weighted imaging, MRI = magnetic resonance imaging.

Ultrasound: A hyperechoic, poorly demarcated lesion was observed in the right hepatic lobe without detectable blood flow signals (Fig. 1A). CT: On unenhanced CT, no abnormal density was identified (Fig. 1B), and the arterial phase revealed a patchy iso- to mildly hypodense lesion in segment VII of the right hepatic lobe (Fig. 1C); during the portal venous phase and delayed phase, the lesion exhibited persistent centripetal enhancement with reduced extent, becoming isodense and poorly demarcated (Fig. 1D–F), measuring approximately 6.3 × 5.0 cm. MRI: On non-contrast MRI, the lesion in segment VII exhibited ill-defined margins with isointensity on T2-weighted imaging (T2WI), diffusion-weighted imaging (DWI) and apparent diffusion coefficient maps, without diffusion restriction, and T1-weighted imaging (T1WI) revealed heterogeneous hypointensity (Fig. 2A–D). Post-contrast imaging demonstrated progressive centripetal enhancement, eventually achieving homogeneity, with delayed-phase imaging displaying iso- to mild hyperintense signals (Fig. 2E–H). Mild displacement of the adjacent vasculature was noted (Fig. 2G), with vascular branches traversing the lesion, but without evidence of vascular invasion (Fig. 2E).

### 2.3. Surgical intervention

The patient underwent partial laparoscopic hepatectomy. Intraoperatively, the lesion was located subcapsularly in the hepatic segment VII without a defined capsule and was completely excised.

### 2.4. Pathological examination and immunohistochemistry

Gross Examination: The excised liver specimen measured 9.0 × 6.5 × 3.8 cm. The cut surface appeared gray-red to gray-yellow and soft, interspersed with multiple hemorrhagic foci. Microscopic Examination: The hepatic lobular architecture was preserved; however, multifocal sinusoidal dilatation, widening, and hemorrhage were noted. Irregular capillary hemangioma-like proliferation and portal fibrosis were observed, with multifocal lymphocytic infiltration and scant plasma cells.

Immunohistochemistry: CD31 (vascular+), CD34 (blood vessel+), ERG (blood vessel+), and D2-40 (lymphatic+). The pathological Diagnosis: Peliosis hepatitis (Fig. 2I).

## 3. Discussion

PH is an infrequent benign vascular anomaly characterized by sinusoidal dilation and the presence of numerous blood-filled cystic cavities within the hepatic parenchyma. The etiopathogenesis remains obscure, although prior studies have postulated potential associations between pharmacological agents, infections, and malignancies.^[1]^ Related pharmacological agents include corticosteroids, immunosuppressants,^[2,3]^ androgens,^[4]^ and oral contraceptives^[5]^; infectious agents include Bartonella henselae,^[6]^ tuberculosis,^[7]^ acquired immunodeficiency syndrome,^[8]^ and syphilis.^[9]^ PH has also been concomitantly observed in hepatocellular carcinoma^[10]^ and hematologic malignancies.^[11]^ However, there is no direct evidence linking these factors to the development of PH. With the widespread application of modern imaging technologies in health screening of asymptomatic populations, an increasing number of focal peliosis hepatis cases without underlying diseases or predisposing factors are being incidentally identified.^[12–14]^ Approximately 20% to 50% of patients show no identifiable underlying associated conditions.^[13]^ In the current case, the patient had no history of exposure to PH-related pharmacological agents, infections (e.g., hepatitis, tuberculosis, and syphilis), human immunodeficiency virus, malignancies, or a familial history of tumors. Consequently, we postulated that this was a primary hepatic lesion.

The pathogenesis of PH may involve damage to the sinusoidal walls by toxic substances, necrosis of hepatocytes leading to cavity formation, or obstruction of sinusoidal outflow.^[15]^ PH can occur at any age, predominantly in adults, with no significant sex predilection.^[16]^ It is typically asymptomatic and is often incidentally detected during routine health examinations or follow-ups for unrelated conditions. In this case, the lesion was identified during a routine ultrasonographic examination, and all laboratory investigations yielded negative results.

Focal PH may present as solitary or multiple lesions, which can be round or irregularly shaped with ill-defined or well-demarcated margins. Radiological manifestations are highly variable and nonspecific, leading to frequent misdiagnoses such as metastatic liver lesions, primary hepatic neoplasms, or abscesses.^[17,18]^ Imaging characteristics depend on factors such as lesion size, temporal stage of intralesional blood, and the presence of thrombosis or hemorrhage.^[19]^ On US, PH appears hypoechoic in patients with fatty liver, but hyperechoic in those with normal liver architecture; hemorrhagic components may contribute to heterogeneous echogenicity.^[18]^ Doppler imaging can reveal vascular patterns within or surrounding a lesion.^[20]^ In the present case, US identified a solitary, slightly hyperechoic lesion with ill-defined margins in the right hepatic lobe, devoid of detectable blood flow signals, which is consistent with existing reports. On unenhanced CT, PH typically manifests as hypodense or isodense lesions with ill-defined borders, although hyperdense lesions have rarely been reported.^[18]^ MRI findings often include homogeneous hypointense signals on T1WI and hyperintense signals on T2WI. Depending on the phase of intralesional hemorrhage or the presence of hemorrhagic necrosis, T1WI may also demonstrate isointense or slightly hyperintense signals.^[21]^ DWI typically shows an unrestricted diffusion. In this case, CT and T2WI revealed isodense or isointense features, likely reflective of the temporal phase of the intralesional blood. DWI and apparent diffusion coefficient signals were isointense relative to the surrounding hepatic parenchyma, indicating an unrestricted diffusion. This feature aids in distinguishing PH from hepatic malignancies such as hepatocellular carcinoma or metastatic lesions, which typically exhibit restricted diffusion. Post-contrast enhancement patterns in focal PH include persistent centrifugal enhancement, progressive centripetal enhancement, homogeneous hyperenhancement, or persistent hypoenhancement.^[22]^ Research indicates that the enhancement pattern of focal PH is contingent on its pathological state. Lesions with fresh, active blood within dilated sinusoids may mimic the intense enhancement observed in hepatocellular carcinoma or hypervascular metastases. Conversely, lesions containing stagnant, aged blood with hepatocellular degeneration or atrophy exhibit persistent hypoenhancement or slow centripetal enhancement.^[19]^ In this case, the lesion demonstrated progressive centripetal enhancement with gradual filling from the late arterial phase to delayed-phase imaging, ultimately achieving iso- to slightly hyperdense or hyperintense signals.

The presence of intralesional traversing vessels may serve as a distinctive feature, characterized by normal intralesional branching vascular structures that maintain continuity with adjacent hepatic or portal veins, suggesting benign growth patterns. Previous studies have reported the absence of a mass effect on the adjacent bile ducts and vascular structures in focal PH.^[18]^ However, mild displacement of the surrounding vasculature was observed in the present case, which is inconsistent with the findings of previous reports. The authors of this study propose that this discrepancy may be attributed to the relatively large size of the lesion coupled with histiocytic proliferation, inflammatory cell infiltration resulting in localized hepatic edema, the spatial relationship between the lesion and adjacent vasculature. In the present case, both intralesional traversing vessels and peripheral vascular displacement were concurrently observed.

In summary, focal PH is a rare clinical entity that poses significant challenges in preoperative diagnosis owing to its diverse radiological manifestations. Key imaging characteristics include unrestricted diffusion on DWI, ill-defined margins, progressive centripetal enhancement, and intralesional traversing vessels. Nevertheless, a definitive diagnosis relies on surgical biopsy and histopathological confirmation.

## Acknowledgments

We would like to express our gratitude to the patient who made this work possible as well as all those who helped us during the writing of this manuscript.

## Author contributions

**Conceptualization:** Zhijian Liu.

**Investigation:** Sumei Gao.

**Resources:** Sumei Gao, Zhijian Liu.

**Writing – original draft:** Tingting Geng.

**Writing – review & editing:** Olena Kovalska, Zhijian Liu.
